# Expression, structure and function analysis of the sperm-oocyte fusion genes *Juno* and *Izumo1* in sheep (*Ovis aries*)

**DOI:** 10.1186/s40104-021-00548-4

**Published:** 2021-03-12

**Authors:** Wenping Hu, Xinlong Dong, Zhilong Tian, Zhuangbiao Zhang, Jishun Tang, Benmeng Liang, Qiuyue Liu, Mingxing Chu

**Affiliations:** 1 Key Laboratory of Animal Genetics and Breeding and Reproduction of Ministry of Agriculture and Rural Affairs, Institute of Animal Sciences, Chinese Academy of Agricultural Sciences, Beijing, 100193 China; 2 Institute of Animal Husbandry and Veterinary Medicine, Anhui Academy of Agricultural Sciences, Hefei, 230031 China; 3 Institute of Genetics and Developmental Biology, the Innovation Academy for Seed Design, Chinese Academy of Sciences, Beijing, 100101 China

**Keywords:** Fertilization, *Izumo1*, *Juno*, Sheep, Single cell RNA-seq

## Abstract

**Background:**

JUNO and IZUMO1 are the first receptor-ligand protein pairs discovered to be essential for sperm-oocyte fusion; their interaction is indispensable for fertilization.

**Methods:**

PCR was used to clone the full-length DNA sequence of the *Juno* gene in sheep. The single nucleotide polymorphism (SNP) loci of *Juno* were genotyped by Sequenom MassARRAY®. PCR combined with rapid amplification of cDNA Ends were used to clone the full-length cDNA sequence of *Juno* and *Izumo1*. Reverse transcriptase-PCR (RT-PCR) and real time-quantitative-PCR (RT-qPCR) were used to analyze the genes’ expression in tissues of sheep, and single cell RNA-seq was used to analyze the genes’ expression in oocytes, granulosa cells and follicular theca of polytocous and monotocous Small Tail Han ewes. Bioinformatics was used to analyze advanced structure and phylogeny of JUNO and IZUMO1 proteins.

**Results:**

The full-length DNA sequence of the *Juno* gene in sheep was cloned and nine SNPs were screened. We found a significant association between the g.848253 C > A locus of *Juno* and litter size of Small Tail Han sheep (*P* < 0.05). The full-length cDNA sequence of *Juno* and *Izumo1* genes from Small Tail Han sheep were obtained. We found a new segment of the *Izumo1* CDS consisting of 35 bp, and we confirmed the *Izumo1* gene has 9 exons, not 8. RT-qPCR showed that *Juno* and *Izumo1* genes were highly expressed in ovarian and testicular tissues, respectively (*P* < 0.01). Single cell RNA-seq showed *Juno* was specifically expressed in oocytes, but not in granulosa cells or follicular theca, while *Izumo*1 displayed little to no expression in all three cell types. There was no difference in expression of the *Juno* gene in oocyte and ovarian tissue in sheep with different litter sizes, indicating expression of *Juno* is not related to litter size traits. Bioinformatic analysis revealed the g.848253 C > A locus of *Juno* results in a nonconservative missense point mutation leading to a change from Phe to Leu at position 219 in the amino acid sequence.

**Conclusions:**

For the first time, this study systematically analyzed the expression, structure and function of *Juno* and *Izumo1* genes and their encoded proteins in Small Tail Han sheep, providing the basis for future studies of the regulatory mechanisms of *Juno* and *Izumo1* genes.

**Supplementary Information:**

The online version contains supplementary material available at 10.1186/s40104-021-00548-4.

## Introduction

Sexual reproduction results in completely new individuals and ensures continuous reproduction for most eukaryotes. Fertilization – the encounter and combination of two morphologically distinct male (sperm) and female (oocyte) germ cells – is the most important part of mammalian sexual reproduction. These two haploid germ cells meet in the reproductive tube of the mother (e.g., the oviduct in mammals), recognize each other, fuse and become a diploid that develops into a genetically unique organism.

Until recently, sperm-oocyte fusion has been a mysterious phenomenon. In 2005, Inoue et al. identified a sperm surface protein, IZUMO1, that is essential for binding to oocytes [1]. Each of the four members of the gene family, *Izumo1*, *Izumo2*, *Izumo3*, and *Izumo4*, share a homologous N-terminal “IZUMO” domain [2].

It took more than 9 years to find the cognate receptor of IZUMO1 on the oocyte cell surface. In the meantime, several receptor proteins were explored as potential recognition factors [3], such as CD9 [4–7]. Although CD9 and IZUMO1 are both essential for fertilization to occur, CD9 was ruled out because IZUMO1 is capable of binding to both wild-type and CD9 deficient oocytes. In 2014, Bianche et al. devised a new technique to identify low-affinity extracellular interactions that allowed them to uncover the IZUMO1 receptor – a GPI-anchored protein called folate receptor 4 (Folr4). Because Folr4 turned out to be the sole receptor for IZUMO1 (and was not able to bind folate after all), it was aptly renamed JUNO, after the Roman goddess of marriage [8]. While the IZUMO1-JUNO adhesion complex is the first ligand-receptor pair known to be essential for fertilization, a recent study found a new protein involved in sperm-oocyte membrane fusion during fertilization, Fertilization Influencing Membrane Protein (FIMP) [9].

The recognition and binding of IZUMO1 and JUNO is an adhesion event, conserved in both human and mouse oocytes [10]. Within 40 min of the first adhesion event between JUNO and IZUMO1, JUNO is completely absent from the oocyte cell surface. During this time, JUNO is packed into vesicles and released outside of the oocyte membrane where it quickly neutralizes other activated sperm. This shedding mechanism ensures the oocyte can only fuse with one sperm, as polyspermy results in a nonviable embryo [11]. Because of the key roles of IZUMO1 and JUNO, their sensitivity is likely to affect the pregnancy rate and sperm-oocyte recognition efficiency of mammals, and overall affect the fecundity of mammals.

The interaction between IZUMO1 and JUNO is conserved in both mammals (e.g., human, mouse, and pig) and nonmammals (e.g., opossum) [8]. Most research on IZUMO1 and JUNO has been conducted in human [12] and mouse, whereas their relevance in sheep reproduction is rarely investigated. There are some research about *Izumo1* in pig [13] and sheep [14]. However, research to date has not addressed JUNO in sheep. In this study, the single nucleotide polymorphisms (SNP) of the *Juno* gene in sheep were identified and genotyped; the association between each SNP and litter size was analyzed. The full-length cDNA sequence of *Juno* and *Izumo1* were cloned from the ovary of Small Tail Han sheep using PCR and RACE technologies, and bioinformatic analyses were carried out to characterize the genes and their hypothetical protein products. The expression of *Juno* and *Izumo1* genes in various tissues were measured with RT-PCR and RT-qPCR. This study provides the basis for further revealing the function and expression regulation of the *Juno* and *Izumo1* genes in sheep, and for further studying the molecular mechanisms regulating reproduction in sheep.

## Materials and methods

### Sequence polymorphism of the sheep *Juno* gene and its association with litter size

#### Materials and main reagents

A total of 760 sheep were selected, including 380 Small Tail Han sheep, a polytocous sheep breed with litter size records, and a total of 380 sheep consisting of five monotocous breeds. There were 100 Sunite sheep, 80 Tan sheep, 39 Suffolk sheep, 30 Dorper sheep and 131 Prairie Tibetan sheep. From each sheep, 10 mL of jugular vein blood was collected and stored at 4 °C after anticoagulation with EDTA.

Small Tail Han sheep were from Chenglian Small Tail Han Sheep Breeding Farm (Yuncheng County, Shandong Province, China) and Shengyi Animal Husbandry Co., Ltd. (Zhangqiu, Shandong Province, China). Sunite sheep were from Minyang Husbandry Co., Ltd. (Wulate Middle Banner in Bayannaoer , Inner Mongolia, China). Tan sheep were from Ningxia Shuomu Yanchi Tan Sheep Breeding Co., Ltd. (Yanchi County, Ningxia, China). Suffolk sheep and Dorper sheep were from Beijing Aoxin Husbandry Co., Ltd. (Beijing, China), and Prairie Tibetan sheep were from Dangxiong County, Tibet, China.

2× Taq PCR Master Mix (MT201-01) was bought from Beijing Biomed Gene Technology Co., Ltd. (Beijing, China). TaKaRa LA Taq® with GC Buffer (RR02AG), TaKaRa MiniBEST Agarose Gel DNA Extraction Kit Ver.4.0 (9762), *E. coli* DH5α competent cells (9057), and pMD™18-T Vector Cloning Kit (6011) were from Takara Bio Co., Ltd. (Dalian, China).

#### DNA extraction and detection

DNA was extracted with a blood genomic DNA extraction kit (Tiangen Biotech Co., LTD, Beijing), according to the manufacturer’s instructions. The purity and concentration of DNA were detected with a NanoDrop 2000 spectrophotometer (Thermo Scientific), and genomic DNA integrity was detected by 1.5% agarose gel electrophoresis. Samples were only considered acceptable if the 260 nm to 280 nm ratio was in the range of 1.8–2.0, the concentration was above 30 ng/mL and the electrophoretic bands were qualified (Fig. S1).

#### Primer design and synthesis

According to the published genome sequence of the sheep *Juno* gene (GenBank accession: NC_019472.2), three primers were designed using Primer Premier 5.0 software. To improve the accuracy of sequencing, primers were designed in segments. The primers were synthesized by Beijing Tianyihuiyuan Biotechnology Co., Ltd. Primer sequence, product size and annealing temperature are shown in Table S1.

#### DNA amplification

Using the extracted blood genomic DNA as templates, the target gene DNA was amplified using the TaKaRa high fidelity LA Taq enzyme system. Each 20 μL reaction contained 0.25 μL TaKaRa LA Taq (5 U/μL), 10 μL 2× GC Buffer I (5 mmol/L Mg^2+^ Plus), 3 μL dNTPs (2.5 mmol/L of each dNTP), 4.25 μL RNase-free ddH_2_0, 0.5 μL forward primer (Table S1), 0.5 μL reverse primer and 1.5 μL blood genomic DNA. The PCR program started with denaturation at 95 °C for 5 min, and was followed by 35 cycles of denaturation at 95 °C for 30 s, annealing at 66 °C for 45 s and extension at 72 °C for 2 min, followed by a final extension at 72 °C for 8 min.

After the reaction, the PCR product was detected by 1.5% agarose gel electrophoresis, and the positive amplification products were cloned and sequenced (Sangon Biotech Co., Ltd., Shanghai, China).

#### SNP loci screening

DNA sequence comparisons and analysis were conducted with DNAMAN 6.0, and peak map judgment was conducted with Chromas Pro 1.5. The DNA sequence of *Juno* was compared with re-sequencing data from our laboratory consisting of 100 individual sheep from 10 different breeds [15]. A loci was considered an SNP if the proportion of different bases at the same locus were more than 30% SNP loci were screened to identify synonymous and non-synonymous mutations.

#### Genotyping

Three pairs of primers were used to screen SNP loci of the *Juno* gene in 60 samples of Small Tail Han sheep and Sunite sheep. Single bands, signifying good specificity in PCR amplification were sequenced bidirectionally. The loci with clear sequence peaks were used for SNP statistics. The SNP loci of the *Juno* gene were genotyped in the blood DNA samples of Small Tail Han sheep, Sunite sheep, Tan sheep, Suffolk sheep, Dorper sheep and Prairie Tibetan sheep with the iPLEX™ assay using the MassARRAY® genotyping system (Sequenom, San Diego, CA, USA). The experimental steps were carried out following the instrument operating guidelines.

#### Statistical analysis

Popgene 32 (version 3.2) and PIC-CALC were used to process the results of genotyping and calculate the heterozygosity (HE) and the polymorphism information content (PIC). The genotype and gene frequencies of SNP loci in polytocous and monotocous breeds were subjected to a chi-square test using SPSS 18.0, and the correlation between genotype frequencies and litter size was analyzed. The relevance analysis model was:

$$ {\mathrm{y}}_{\mathrm{i}\mathrm{jkl}}=\upmu +{\mathrm{G}}_{\mathrm{i}}+{\mathrm{P}}_{\mathrm{j}}+{\mathrm{S}}_{\mathrm{k}}+{\mathrm{e}}_{\mathrm{i}\mathrm{jkl}} $$

Where, *y* is the phenotypic value of the litter size, *μ* is the population mean, *G*_*i*_ is the genotype effect, *P*_*j*_ is parity effect, _*Sk*_ is the field effect, and *e*_*ijkl*_ is the random residual effect of each observation. Assuming each observation was independent, we used a N (0, σ^2^) distribution.

### cDNA cloning and differential expression analysis of *Juno* and *Izumo1* genes in sheep

#### Materials and main reagents

Three male Small Tail Han sheep, three female Small Tail Han sheep, three male Sunite sheep, and three female Sunite sheep in good health and with the same bodyweight and sexual maturity were selected for euthanasia,sheep and Sunite sheep for euthanasia. Sixteen types of tissues, including gonadal (testis, epididymis, sperm duct/ovary, uterus, oviduct), pineal gland, hypothalamus, pituitary, cerebellum, heart, liver, spleen, lung, kidney, skeletal muscle, duodenum, subcutaneous abdominal fat and adrenal gland were collected quickly and put into a pre-labeled 1.8 mL RNase-Free cryopreservation tube. The tubes were immediately placed in liquid nitrogen and transferred to a cryogenic refrigerator (− 80 °C) for storage.

TRIZOL® LS Reagent and SYBR® Safe DNA gel stain (S33102) were bought from Invitrogen, ThermoFisher Scientific, CN. Total RNA Extraction Kit for Animal Tissues (DP431) was bought from Tiangen Biotech Co., LTD (Beijing). 2× Taq PCR Master Mix (MT201-01) was bought from Biomed Gene Technology Co., LTD. TaKaRa LA Taq® with GC Buffer (RR02AG), PrimeScript™ RT reagent kit (Perfect Real Time) (RR037A), TaKaRa MiniBEST Agarose Gel DNA Extraction Kit Ver.4.0 (9762), SMARTer® RACE 5′/3′Kit (634858), *E. coli* DH5α competent cells (9057), pMD™18-T Vector Cloning Kit (6011), PrimeScript® RT reagent Kit Perfect Real-Time (RR037A) and SYBR® Premix Ex Taq™ II (Tli RNaseH Plus) (RR820A) were bought from Takara Bio Co., Ltd. (Dalian, China).

#### cDNA cloning of *Juno* and *Izumo1* genes

- Extraction of total RNA from samples

Samples from different parts of each sheep were ground in liquid nitrogen, and then total RNA was extracted with the Total RNA Extraction Kit for Animal Tissues (DP431). RNA integrity was assessed by 1% agarose gel electrophoresis, and RNA concentration and purity were measured by nanodrop spectrophotometry. Only RNA samples displaying a 260 nm/280 nm ratio between 1.8 and 2 were used in this study. The product was stored in a − 80 °C cryogenic freezer until further use.

- Coding sequence (CDS) cloning of *Juno* and *Izumo1* genes

Total RNA of the ovaries and testes of Small Tail Han sheep was subjected to RT-PCR with the PrimeScript™ RT reagent kit. The reverse transcription products were stored at − 20 °C. According to the predicted *Juno* and *Izumo1* genes sequences (NCBI reference sequences: XM_015100756.1 and NM_001164492.1), the amplification primers of *Juno* and *Izumo1* were designed with Primer Premier 5.0 software (Table S2).

The cDNA from ovaries and testes of Small Tail Han sheep obtained by RT-PCR was used as templates to clone the *Juno* and *Izumo1* genes using the TaKaRa LA Taq® with GC Buffer (RR02AG) enzyme system. Each 20 μL reaction contained 1 μL cDNA, 0.5 μL forward primer (Table S2), 0.5 μL reverse primer, 10 μL 2× Taq PCR Master Mix and 8 μL ddH_2_O. The PCR program started with denaturation at 95 °C for 5 min, followed by 35 cycles of denaturation at 95 °C for 30 s, annealing at 62 °C for 30 s and extension at 72 °C for 5 s, followed by a final extension at 72 °C for 8 min. The amplified products were detected by 1.5% agarose gel electrophoresis. The target fragments were recovered using the TaKaRa MiniBEST Agarose Gel DNA Extraction Kit Ver.4.0 (9762), ligated into a pMD18-T (TaKaRa) vector, and transformed into DH5α competent cells. Ampicillin resistant colonies were screened by colony PCR and DNA fragments were sent to Sangon Biotech Co., Ltd. (Shanghai) for sequencing.

- 3′ /5′ Rapid amplification of cDNA ends (RACE) of *Juno* and *Izumo1* cDNA

Total RNA of ovaries and testes of Small Tail Han sheep were used as templates, and a SMARTer® RACE 5′/3′Kit was used for reverse transcription. According to the sequence of the CDS region obtained by sequencing, 5′ and 3′ RACE specific primers were designed according to the requirements of the SMARTer® RACE 5′/3′ Kit. Using the reversed first chain of 3′ RACE and 5′ RACE as templates, the 3′ and 5′ ends of the *Juno* and *Izumo1* genes from sheep were amplified by Touch Down PCR using specific primers 3’GSP and 5’GSP, and a universal primer UPM (Table S2).

#### Expression of *Juno* and *Izumo1* genes in different tissues

- RT-PCR of *Juno* and *Izumo1* genes

Using cDNA obtained from 16 tissues of Small Tail Han sheep and Sunite sheep as templates, Juno-RT primers, Izumo1-RT primers and reference gene (β-actin) primers, PCR amplification was conducted. Each 20 μL reaction consisted of 10 μL 2× Taq PCR Master Mix, 8 μL ddH_2_O, 0.5 μL forward primer, 0.5 μL reverse primer and 1 μL cDNA. The PCR program began with denaturation at 95 °C for 5 min, followed by 25 cycles of denaturation at 95 °C for 30 s, annealing at 62 °C for 30 s and extension at 72 °C for 5 s, followed by a final extension at 72 °C for 8 min. The amplified products were analyzed by agarose gel electrophoresis. The quality of the cDNA obtained from reverse transcription was amplified with the primers of the reference gene β-actin to verify that it could be used for subsequent experiments.

- RT-qPCR of the *Juno* and *Izumo1* genes in sheep

Using cDNA obtained from 16 tissues of Small Tail Han sheep and Sunite sheep as templates, RT-qPCR was conducted. Each 20 μL reaction contained 10 μL SYBR Premix Ex Taq II, 6.4 μL RNase-Free ddH_2_O, 0.8 μL forward primer, 0.8 μL reverse primer and 2 μL cDNA. The RT-qPCR reaction started with denaturation at 95 °C for 5 s, followed by 40 cycles of denaturation at 95 °C for 5 s and annealing at 60 °C for 30 s. Each sample was run in triplicate and β-actin was used as the reference gene. The relative expression of each target gene was calculated with the 2^−△△Ct^ method. The data were analyzed with the statistical software SPSS 18.0, and the statistical data were reported as the mean ± the standard deviation. One-way ANOVA was used to detect differences in expression among different tissues, and the Student’s *t*-test was used to compare the differences in expression among the same tissues. *P* < 0.05 indicated a significant difference.

#### Single cell expression of genes by single cell RNA-seq

Polytocous and monotocous Small Tail Han ewes 3 years of age and with three or higher parity records were selected for single cell gene expression analysis. There were five ewes in each group. A controlled internal drug release (CIDR) device (InterAg company, New Zealand) was used to synchronize the estrus cycles of ewes by administering 300 mg progesterone. After 12 d, the CIDR device was removed. Forty-five h after the CIDR device was removed, the ewes were operated on to collect oocytes, granulosa cells and follicular theca.

The Smart-Seq2 method was used to directly measure gene expression in single cells [16]. A Qubit® 3.0 Fluorometer (Life Technologies, CA, USA) and an Agilent 2100 High Sensitivity DNA Assay Kit (Agilent Technologies, CA, USA) were used to detect the amplified cDNA concentration and fragment distribution. After that, single-cell cDNA libraries were constructed, and the length distribution of the libraries was detected using an Agilent 2100/LabChip GX Touch. After meeting the length distribution requirements, qPCR was used to accurately quantify the effective concentration of the cDNA in the libraries (more than 10 nmol/L). After the effective concentration of the libraries was qualified, the PE150 double terminal sequencing program was run on the HiSeq sequencing platform for single cell RNA-seq analysis.

Raw reads from Illumina sequencing were filtered to get clean reads for subsequent analyses. With the Hierarchical Graph FM index (HGFM) as the core retrieval method, HiSAT2 was used to filter the RNA-seq data of each sample with the sheep reference genome (Ovis_aries Oar_v3.1pep_all.fasta). Using StringTie software, RNA-seq comparison results were quickly assembled into transcripts and gene expression levels were predicted.

### Bioinformatic analysis of *Juno* and *Izumo1* cDNAs

The sequencing results were subjected to a BLAST search (NCBI, http://blast.ncbi.nlm.nih.gov/Blast.cgi) to retrieve homologous gene sequences, and DNAMAN software was used to splice the sequences into the full-length cDNA sequence of each gene. Open reading frame (ORF) Finder (http://www.ncbi.nlm.nih.gov/projects/gorf/) was used to determine the ORF and predict the amino acid sequence. ProtParam (http://web.expasy.org/protparam/) was used to predict the physical parameters of each protein such as molecular weight. The hydrophilicity and hydrophobicity of JUNO and IZUMO1 of Small Tail Han sheep were analyzed using ProtScale (https://web.expasy.org/protscale/). Prediction of the secondary structure of each protein and its variants was carried out using PredictProtein (https://www.predictprotein.org/). Phyre2 was used to predict the protein signal peptide, protein transmembrane region and protein tertiary structure. Multiple amino acid sequences were compared using DNAMAN software, and a phylogenetic tree was constructed using the Neighbor-Joining method in MEGA7 software (reference).

## Results

### Polymorphism of the *Juno* gene in sheep and its association with litter size

#### DNA amplification

Two complete DNA sequences of the *Juno* gene in Small Tail Han sheep and Sunite sheep were successfully obtained. The full-length DNA sequences of the *Juno* gene in Small Tail Han sheep and Sunite sheep were 2167 bp and 2168 bp, respectively, and those two sequences were more than 95% identity. The length of *Juno* in Small Tail Han sheep and Sunite sheep is similar to the full-length DNA sequence of the *Juno* gene in Texel sheep (2166 bp, NCBI Reference Sequence: NC_040266.1).

#### SNPs identification based on whole genome re-sequencing results

Based on whole genome re-sequencing data from our laboratory consisting of 100 individual sheep from 10 different breeds [15], all SNPs near this region of *Juno* gene were checked, nine SNP loci of *Juno* were identified: g.844598A > G, g.846206C > T, g.846268A > G, g.847219G > C, g.847810C > G, g.847814C > T, g.847830T > C, g.847927A > C and g.848253C > A. Among them, eight were in exons and one was in a 5′ UTR. Of the 8 exonic SNPs, five were missense mutations and 3 were synonymous mutations. There were three T/C transitions, two A/G transitions, two C/A transversions and two G/C transversions.

#### Association between SNP loci of *Juno* and litter size in sheep

According to the data obtained from genotyping, nine SNP loci were preliminarily screened, and SNP loci with no difference in the allele frequencies between monotocous and polytocous populations were excluded. Seven SNP loci were further analyzed (Table 1). Genotyping showed that there were three genotypes at *Juno* gene loci g.844598A > G, g.847830T > C, g.847927A > C, g.846268A > G and g.847810C > G in monotocous and polytocous breeds, two genotypes (GG and CG) at g.847219G > C in the monotocous breed and three genotypes (GG, CG and CC) in polytocous breed (Table 1). There were CC and CA genotypes in monotocous breeds and CC, CA and AA genotypes in polytocous breeds at the g.848253C > A locus. Among them, the genotype frequencies of *Juno* gene loci g.847830T > C, g.846268A > G and g.847219G > C were significantly different between monotocous and polytocous sheep populations (*P* < 0.01). The allele frequencies of *Juno* gene on loci g.847927A > C, g.846268A > G and g.847219G > C were significantly different between monotocous and polytocous sheep populations (*P* < 0.01).

**Table 1:** Genotype and allele frequencies of seven SNPs at *Juno* gene in monotocous and polytocous sheep

Locus	Genotype	Genotype frequency in polytocous sheep	Genotype frequency in monotocous sheep	Chi-square test (***P***-value)	Allele	Allele frequency in polytocous sheep	Allele frequency in monotocous sheep	Chi-square test (***P***-value)
g.844598A > G	GG	0.31	0.36	0.28	G	0.55	0.58	0.25
	GA	0.48	0.43		A	0.45	0.42	
	AA	0.21	0.21					
g.847830T > C	CC	0.53	0.60	0.01	C	0.72	0.75	0.28
	CT	0.39	0.29		T	0.28	0.25	
	TT	0.08	0.11					
g.847927A > C	CC	0.20	0.16	0.07	C	0.37	0.31	0.01
	CA	0.33	0.29		A	0.63	0.69	
	AA	0.47	0.55					
g.846268A > G	GG	0.36	0.28	0.00	G	0.59	0.50	0.00
	GA	0.47	0.44		A	0.41	0.50	
	AA	0.17	0.28					
g.847219G > C	GG	0.74	0.86	0.00	G	0.85	0.93	0.00
	CG	0.22	0.13		C	0.15	0.07	
	CC	0.03	0.00					
g.847810C > G	CC	0.71	0.73	0.21	C	0.85	0.85	0.98
	GC	0.28	0.25		G	0.15	0.15	
	GG	0.01	0.03					
g.848253C > A	CC	0.84	0.88	0.16	C	0.91	0.94	0.07
	CA	0.15	0.12		A	0.09	0.06	
	AA	0.01	0.00					

*P* values < 0.01 are considered statistically significant

We analyzed the polymorphism distributions of *Juno* gene in six sheep breeds (Table 2). The polymorphic information contents (*PIC*) of loci g.847219G > C and g.848253C > A were low in each of the six sheep breeds (*PIC* < 0.25), whereas the *PIC* in loci g.844598A > G, g.847830T > C, g.847927A > C, g.846268A > G and g.847810C > G were both low (*PIC* < 0.25) and moderate (0.25 < *PIC* < 0.5). Among seven polymorphic loci, only g. 847810C > G was under Hardy-Weinberg equilibrium in all six sheep breeds (Table 2).

**Table 2:** The genetic polymorphism information of seven SNPs at *Juno* gene in six sheep breeds

Allele	Breeds	Genotype frequency			Allele frequency		PIC	HE	NE	Chi-square test (*P*-value)
g.844598A > G		GG	GA	AA	G	A				
	Small Tail Han sheep	0.31	0.48	0.21	0.55	0.45	0.37	0.50	1.98	0.49
	Tan sheep	0.23	0.53	0.24	0.49	0.51	0.37	0.50	2.00	0.57
	Sunite sheep	0.24	0.47	0.29	0.47	0.53	0.37	0.50	1.99	0.63
	Suffolk sheep	0.08	0.15	0.77	0.15	0.85	0.23	0.26	1.35	0.01
	Dorper sheep	0.07	0.45	0.48	0.29	0.71	0.33	0.41	1.71	0.66
	Prairie Tibetan sheep	0.33	0.33	0.33	0.50	0.50	0.38	0.50	2.00	0.05
g.847830T > C		CC	CT	TT	C	T				
	Small Tail Han sheep	0.53	0.39	0.08	0.72	0.28	0.32	0.40	1.67	0.55
	Tan sheep	0.55	0.38	0.07	0.74	0.26	0.31	0.38	1.62	0.94
	Sunite sheep	0.48	0.34	0.18	0.65	0.35	0.35	0.46	1.84	0.01
	Suffolk sheep	0.92	0.08	0.00	0.96	0.04	0.07	0.08	1.08	0.80
	Dorper sheep	0.97	0.03	0.00	0.98	0.02	0.03	0.03	1.03	0.93
	Prairie Tibetan sheep	0.48	0.27	0.24	0.62	0.38	0.36	0.47	1.89	0.02
g.847927A > C		AA	CA	CC	A	G				
	Small Tail Han sheep	0.47	0.33	0.20	0.63	0.37	0.36	0.46	1.87	0.00
	Tan sheep	0.52	0.29	0.19	0.66	0.34	0.35	0.45	1.80	0.00
	Sunite sheep	0.42	0.35	0.23	0.60	0.41	0.37	0.48	1.93	0.01
	Suffolk sheep	0.87	0.13	0.00	0.94	0.06	0.11	0.12	1.14	0.67
	Dorper sheep	0.97	0.03	0.00	0.98	0.02	0.03	0.03	1.03	0.93
	Prairie Tibetan sheep	0.43	0.26	0.31	0.56	0.44	0.37	0.49	1.97	0.00
g.846268A > G		AA	GA	GG	A	G				
	Small Tail Han sheep	0.17	0.47	0.36	0.41	0.59	0.37	0.48	1.93	0.59
	Tan sheep	0.11	0.51	0.38	0.37	0.63	0.36	0.47	1.87	0.37
	Sunite sheep	0.21	0.48	0.30	0.45	0.55	0.37	0.50	1.98	0.83
	Suffolk sheep	0.77	0.13	0.10	0.83	0.17	0.24	0.28	1.38	0.00
	Dorper sheep	0.40	0.50	0.10	0.65	0.35	0.35	0.46	1.83	0.59
	Prairie Tibetan sheep	0.28	0.44	0.28	0.50	0.50	0.38	0.50	2.00	0.50
g.847219G > C		GG	CG	CC	G	C				
	Small Tail Han sheep	0.74	0.22	0.03	0.85	0.15	0.22	0.25	1.33	0.05
	Tan sheep	0.01	0.26	0.73	0.14	0.86	0.22	0.25	1.33	0.55
	Sunite sheep	0.00	0.11	0.89	0.06	0.94	0.10	0.10	1.12	0.56
	Suffolk sheep	0.00	0.03	0.97	0.01	0.99	0.03	0.03	1.03	0.93
	Dorper sheep	0.00	0.13	0.87	0.07	0.93	0.12	0.12	1.14	0.70
	Prairie Tibetan sheep	0.00	0.11	0.89	0.06	0.94	0.10	0.10	1.12	0.72
g.847810C > G		CC	GC	GG	C	G				
	Small Tail Han sheep	0.71	0.28	0.01	0.85	0.15	0.22	0.26	1.34	0.08
	Tan sheep	0.64	0.30	0.06	0.79	0.21	0.28	0.33	1.50	0.35
	Sunite sheep	0.75	0.25	0.00	0.88	0.13	0.19	0.22	1.28	0.15
	Suffolk sheep	0.85	0.13	0.03	0.91	0.09	0.15	0.16	1.20	0.18
	Dorper sheep	0.53	0.40	0.07	0.73	0.27	0.31	0.39	1.64	0.90
	Prairie Tibetan sheep	0.78	0.22	0.00	0.89	0.11	0.18	0.20	1.25	0.45
g.848253C > A		AA	CA	CC	A	C				
	Small Tail Han sheep	0.01	0.15	0.84	0.09	0.91	0.14	0.16	1.18	0.38
	Tan sheep	0.01	0.25	0.74	0.14	0.86	0.21	0.24	1.31	0.63
	Sunite sheep	0.00	0.10	0.90	0.05	0.95	0.09	0.10	1.11	0.60
	Suffolk sheep	0.00	0.00	1.00	0.00	1.00	0.00	0.00	1.00	NA
	Dorper sheep	0.00	0.00	1.00	0.00	1.00	0.00	0.00	1.00	NA
	Prairie Tibetan sheep	0.00	0.11	0.89	0.06	0.94	0.10	0.10	1.12	0.72

*P* > 0.05 indicates the locus was under Hardy-Weinberg equilibrium. *P* < 0.05 indicates the locus was not under Hardy-Weinberg equilibrium

Association analysis between each of the seven polymorphic loci and litter size of Small Tail Han sheep revealed a significant association between locus g.848253C > A and litter size in different parities (*P* < 0.05) (Table 3). Overall, the litter size with the CC genotype was greater than that with AA genotype at different parities, but there was no significant difference on allele frequencies between the CC and CA genotypes. Small Tail Han sheep is a polytocous breed with an average litter size of 2.67. Taking the third parity of Small Tail Han sheep as an example, the average litter size of the CC genotype was 2.88 (±0.10), while that of the AA genotype was 1.50 (±0.33).

**Table 3:** Association of different SNP genotypes of the *Juno* gene with litter size in Small Tail Han sheep

Locus	Genotype	No. of the 1st parity	Litter size of the 1st parity	No. of the 2nd parity	Litter size of the 2nd parity	No. of the 3rd parity	Litter size of the 3rd parity
g.844598A > G	AA	74	2.15 ± 0.09	78	2.32 ± 0.10	21	3.00 ± 0.22
	GA	159	2.06 ± 0.06	175	2.24 ± 0.06	69	2.77 ± 0.12
	GG	102	2.26 ± 0.08	114	2.19 ± 0.08	36	2.98 ± 0.17
g.847830T > C	CC	175	2.19 ± 0.06	191	2.21 ± 0.06	21	2.87 ± 0.12
	CT	131	2.07 ± 0.07	142	2.35 ± 0.07	36	2.93 ± 0.14
	TT	28	2.10 ± 0.15	31	2.32 ± 0.15	69	2.57 ± 0.39
g.847927A > C	AA	158	2.18 ± 0.06	171	2.23 ± 0.07	62	2.94 ± 0.13
	CA	110	2.10 ± 0.07	76	2.28 ± 0.10	16	2.56 ± 0.25
	CC	67	2.11 ± 0.10	119	2.32 ± 0.08	49	2.94 ± 0.15
g.846268A > G	AA	57	2.15 ± 0.10	64	2.23 ± 0.11	25	3.00 ± 0.20
	GA	159	2.17 ± 0.06	174	2.25 ± 0.07	66	2.89 ± 0.13
	GG	123	2.11 ± 0.07	133	2.29 ± 0.08	39	2.74 ± 0.16
g.847219G > C	CC	12	2.33 ± 0.23	13	2.00 ± 0.24	3	2.33 ± 0.59
	CG	74	2.14 ± 0.09	83	2.21 ± 0.09	25	2.70 ± 0.20
	GG	255	2.12 ± 0.05	277	2.29 ± 0.05	102	2.92 ± 0.10
g.847810C > G	CC	231	2.14 ± 0.05	260	2.22 ± 0.05^a^	87	2.85 ± 011
	GC	98	2.15 ± 0.08	100	2.39 ± 0.09^a^	36	2.97 ± 0.17
	GG	4	2.25 ± 0.39	4	1.50 ± 0.43^b^	2	2.50 ± 0.73
g.848253C > A	AA	4	1.25 ± 0.39^a^	4	1.25 ± 0.43^a^	4	1.50 ± 0.33^a^
	CA	48	2.13 ± 0.11^b^	54	2.17 ± 0.12^b^	10	2.91 ± 0.31^b^
	CC	281	2.13 ± 0.05^b^	307	2.28 ± 0.05^b^	116	2.88 ± 0.10^b^

^a, b^: Different lowcases indicate statistically significant differences (*P* < 0.05)

### cDNA cloning and expression profiles of *Juno* and *Izumo1* genes in sheep

#### CDS cloning of *Juno* and *Izumo1*

The RT-PCR products of the *Juno* and *Izumo1* genes from Small Tail Han sheep were 463 bp and 895 bp (Fig. S2), respectively. The *Juno* gene from Small Tail Han sheep was 98% identity to the gene from goat (*Capra hircus*, GenBank accession: XM_018058935.1) and 95% identity to the gene from Texel sheep (*Ovis aries*, GenBank accession: XM_015100756.1). The *Izumo1* gene from Small Tail Han sheep was 95% identity to the gene from goat (*Capra hircus*, GenBank accession: NM_001287235.1) and 99% identity to the gene from Texel sheep (*Ovis aries*, GenBank accession: NM_001164492.1). The high similarity of Small Tail Han sheep *Izumo1* and *Juno* gene sequences indicated that those genes are very conservative among Bovidae.

#### Full-length amplification of *Juno* and *Izumo1* cDNA

Using the obtained partial RT-PCR product of the *Juno* gene of Small Tail Han sheep as template, a 572-bp fragment was obtained by 3′ RACE, and 658 bp and 518 bp fragments were obtained by 5′ RACE (Fig. S2). The full sequence of the *Juno* gene was 1131 bpwhich contained a  50-bp 5′ UTR, a 349-bp 3′ UTR and a 732-bp CDS. The gene contained 4 exons and 3 introns, encoding a protein comprised of 243 amino acids.

Using the partial RT-PCR product of the *Izumo1* gene of Small Tail Han sheep as the template, 290 bp, and 1010 bp were obtained by 3′ RACE and 5′ RACE, respectively (Fig. S2). The *Izumo1* gene was 1565 bp, consisting of 9 exons and 8 introns. It contained a 433-bp 5′ UTR, a 97-bp 3′ UTR and a 1035-bp CDS. The initial codon ATG began at nt 434 and the termination codon TAG began at nt 1466, encoding a protein 344 amino acids in length.

**Fig. 1:**
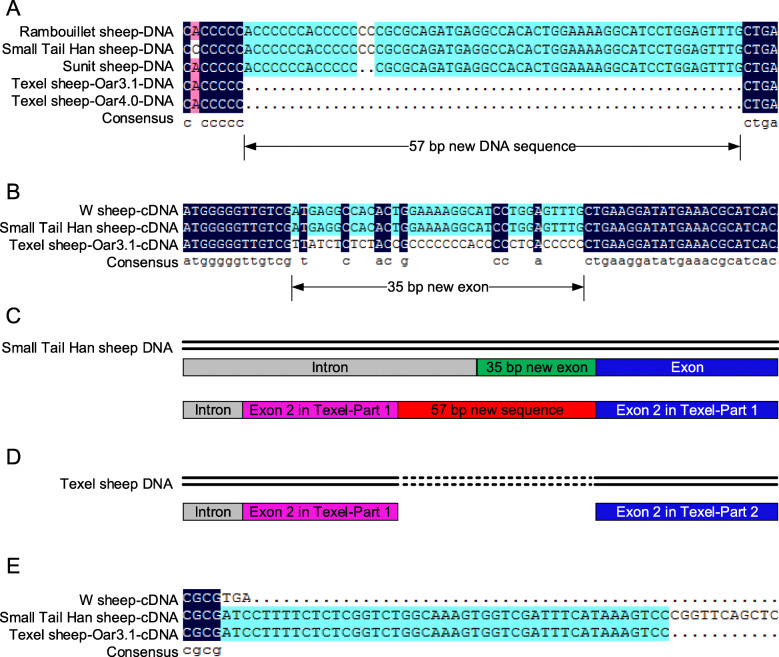
Sequence alignment results of *Izumo1* DNA and full-length cDNA. **a** Sequence alignment results of *Izumo1* DNA. Rambouillet sheep-DNA means *Izumo1* DNA sequence of Rambouillet sheep (GenBank:NC_040265.1); Small Tail Han sheep-DNA means *Izumo1* DNA sequence of Small Tail Han sheep, which was we cloned [17]; Sunit sheep-DNA means *Izumo1* DNA sequence of Sunit sheep, which was we cloned [17]. Texel sheep-Oar3.1-DNA means *Izumo1* DNA sequence of Texel sheep (Oar_v3.1: NC_019471.1); Texel sheep-Oar4.0-DNA means *Izumo1* DNA sequence of Texel sheep (Oar_v4.0: NC_019471.2). **b** Sequence comparison results of exon 2 of *Izumo1* cDNA. W sheep-cDNA means *Izumo1* cDNA sequence from unKnow breed of sheep (GenBank:NM_001164492.1); Small Tail Han sheep-cDNA means *Izumo1* cDNA sequence that we cloned in this research; Texel sheep-Oar3.1-cDNA means CDS of *Izumo1* in Texel Sheep (Ensembl: ENSOART00000013198.1 IZUMO1–201 cDNA:protein_coding). **c** Part of the *Izumo1* DNA of Small Tail Han sheep. The red box reprent the 57 bp new sequence in Small Tail Han sheep DNA which is not exit in Texel sheep; The green box reprent the 35 bp new sequence which belong to Exon2 of *Izumo1* in Small Tail Han sheep. **d** Part of the *Izumo1* DNA of Texel sheep; **e** Sequence alignment of termination codon region of *Izumo1* cDNA. The left tag is the same as (**b**)

We previous reported that the DNA sequence of *Izumo1* gene from Texel sheep (Oar_v4.0: NC_019471.2 and Oar_v3.1: NC_019471.1) was 57 shorter than the corresponding genes in Small Tail Han sheep [17]. A 35-bp sequence 5'-ATGAGGCCACACTGGAAAAGGCATCCTGGAGTTTG-3' derived from this 57 bp DNA sequence. The 35 bp insertionfrom 239–273 bp of the CDS sequence of the *Izumo1* gene in Small Tail Han sheep did not exist in the CDS of Texel sheep (Fig. 1).

After sequence alignment, it demonstrated that 35 bp of the extra 57 bp belongs to exon 2 (Fig. 1c, Exon 2 in Small Tail Han sheep Part 1), while the other 22 bp belongs to intron 1 (Fig. 1c) in Small Tail Han sheep. We further compared the CDS of *Izumo1* in Texel Sheep (Ensembl: ENSOART00000013198.1 IZUMO1-201 cDNA:protein_coding) and found that the second half of exon 2 (exon 2 in Texel sheep-Part 2) was the same as that of Small Tail Han sheep (exon 2 in Small Tail Han sheep-Part 2). But the first half sequence of exon 2 of Texel Sheep (exon 2 in Texel sheep-Part 1) was in the intron 1 region of the *Izumo1* genome of Small Tail Han sheep (Fig. 1b, c, d). We speculated that the 57 bp DNA sequence of Small Tail Han sheep may lead to different transcript splicing patterns. In addition, we compared the *Izumo1* DNA sequence of Rambouillet sheep to that from Small Tail Han sheep (Fig. 1a, GenBank accession: NC_040265.1). The *Izumo1* DNA sequence of Rambouillet sheep was highly similar to that of Small Tail Han sheep and Sunite sheep and it contained a similar insert. We suspect the CDS of Rambouillet sheep may also be similar to that of Small Tail Han sheep and Sunite sheep.

In addition, we found that there were only eight exons in the sheep *Izumo1* CDS in the NCBI database (GenBank accession: NM_001164492.1), and the CDS region of the sequence terminated early at exon 8. The full-length *Izumo1* cDNA sequence of Small Tail Han sheep we obtained had nine complete exons (Fig. 1e). The cDNA sequence of *Izumo1* of Texel Sheep provided in the Ensembl database (Ensembl: ENSOART00000013198.1 IZUMO1-201 cDNA:protein_coding) was incomplete, and the position of the terminator was not indicated. Therefore, this study provides the first full-length *Izumo1* cDNA sequence of sheep.

#### Differential expression analysis of *Juno* and *Izumo1* genes in tissues of sheep

**Fig. 2:**
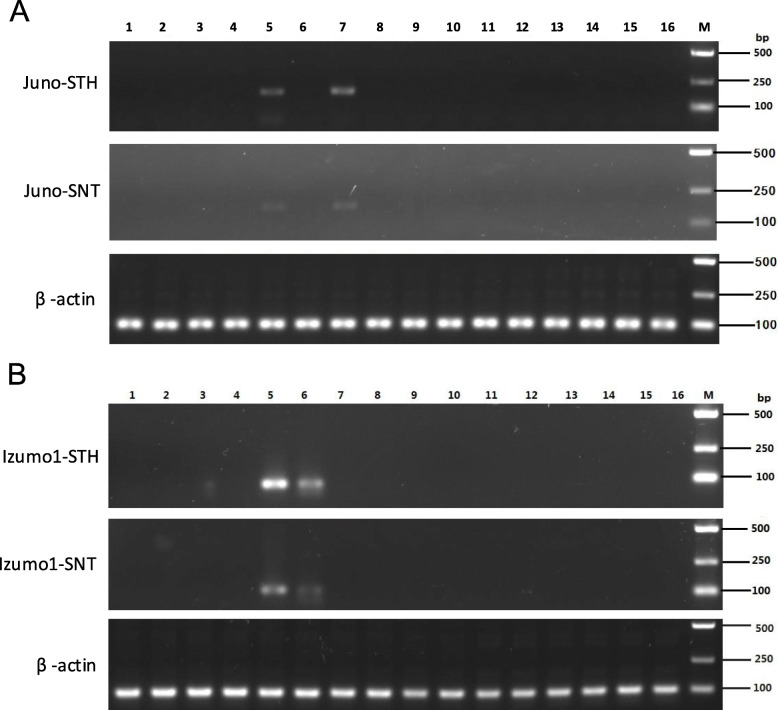
Semi-quantitative RT-PCR of the *Juno* and *Izumo1* genes from Small Tail Han sheep (STH) and Sunite sheep (SNT). **a** *Juno* gene expression in different tissues, 1: pineal gland, 2: hypothalamus, 3: pituitary gland, 4: cerebellum, 5: ovary, 6: uterus, 7: oviduct, 8: heart, 9: liver, 10: spleen, 11: lung, 12: kidney, 13: skeletal muscle, 14: duodenum, 15: subcutaneous fat, 16: adrenal gland and M: DL5000DNA Marker. **b** *Izumo1* gene expression in different tissues, 1: pineal gland, 2: hypothalamus, 3: pituitary gland, 4: cerebellum, 5: testis, 6: epididymis, 7: ductus deferens, 8: heart; 9: liver, 10: spleen, 11: lung, 12: kidney, 13: skeletal muscle, 14: duodenum, 15: subcutaneous fat, 16: adrenal gland and M: DL5000 DNA Marker

Semi-quantitative RT-PCR results showed that the *Juno* gene was expressed in ovaries and oviducts both in Small Tail Han sheep and Sunite sheep, but not in the other 14 tissues tested (Fig. 2a). The *Izumo1* gene was expressed only in the testes and epididymis of Small Tail Han sheep and Sunite sheep, but not in the other 14 tissues tested. The expression level of *Izumo1* in the testes of the two sheep breeds was higher than that in the epididymis (Fig. 2b).

**Fig. 3:**
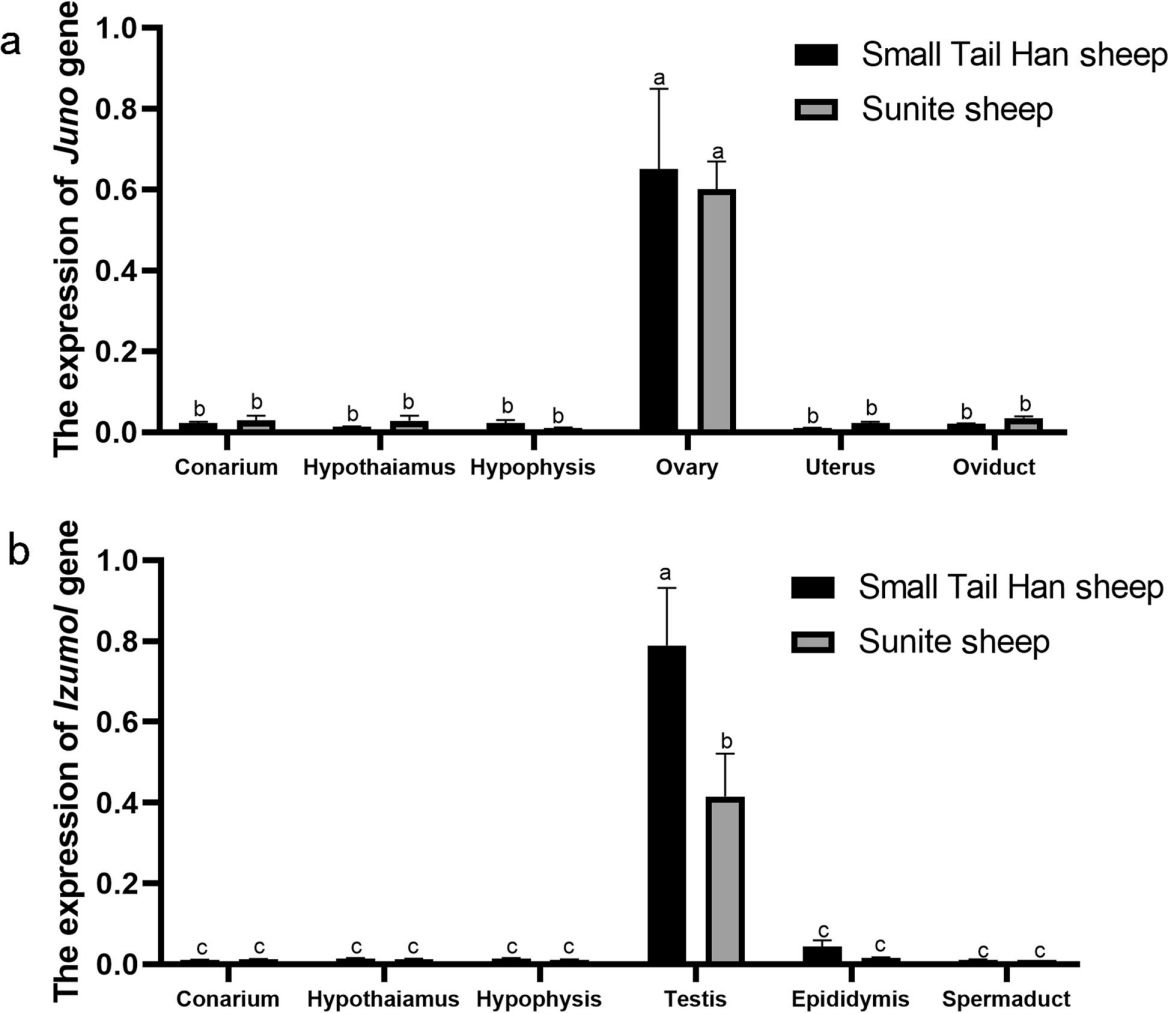
Expression of *Juno* and *Izumo1* genes from Small Tail Han sheep and Sunite sheep in the reproductive gonad axis. Samples denoted by the same superscript did not have different expression (*P* > 0.05). Samples denoted by different superscripts were remarkably different (*P* < 0.05)

The expression of *Juno* and *Izumo1* in different tissues of the reproductive gonad axis of sheep was measured by RT-qPCR (Fig. 3). *Juno* expression was significantly higher in the ovaries than all other tissues (*P* < 0.01), but there was no significant difference between Small Tail Han sheep and Sunite sheep (*P* > 0.05). Expression of *Izumo1* was higher in the testes than all other tissues (*P* < 0.01), but there was no difference between expression in the other tissues. Unlike *Juno*, the expression of *Izumo1* in the testes of Small Tail Han sheep, a polytocous breed, was significantly higher than that in Sunite sheep, a monotocous breed (*P* < 0.01). Although the expression of *Izumo1* in other tissues of Small Tail Han sheep was higher than that of Sunite sheep, the difference was not significant (*P* > 0.05).

#### Gene expression profiles at single cell levels

**Fig. 4:**
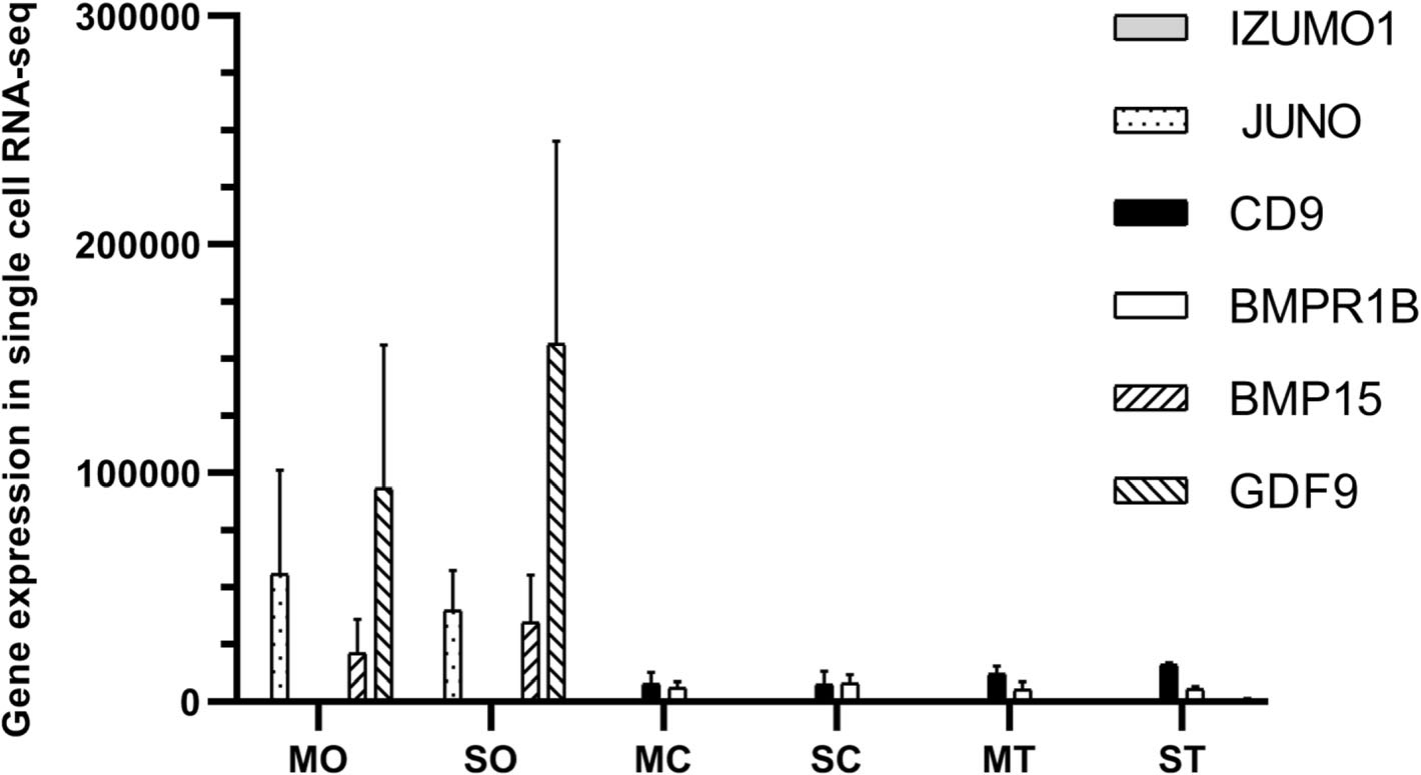
Expression of genes by single cell RNA-seq. MO: oocyte of polytocous sheep, SO: oocyte of monotocous sheep, MC: granulosa cell of polytocous sheep, SC: granulosa cell of monotocous sheep, MT: follicular theca of polytocous sheep and ST: follicular theca of monotocous sheep

There are many types of cells in ovarian tissue, and there is heterogeneity between cells. To further explore the expression and function of the *Juno* gene in different types of cells in ovarian tissue, we collected single cells, including oocytes, granulosa cells and follicular theca of polytocous and monotocous Small Tail Han ewes, and sequenced them by single cell RNA-seq. The expression levels of *Juno*, *BMP15* and *GDF9* were very high in oocytes, but very low in granulosa cells and follicular theca (Fig. 4). However, expression of *BMPR1B* and *CD9* was high in granulosa cells and follicular theca but very low in oocytes. At the same time, we found that *Izumo1* had barely expression in all three cell types. The results showed that in ovarian tissue, *Juno* was specifically expressed in oocytes, while *Izumo1* was specifically expressed in testes, consistent with the function of the two genes. In addition, there was no difference in the expression of *Juno* in oocytes or ovaries between polytocous and monotocous Small Tail Han sheep, suggesting the difference in litter size is not caused by the level of *Juno* expression, but is possibly due to some other factor.

### Bioinformatic analysis of JUNO and IZUMO1 proteins

#### Physicochemical properties of JUNO and IZUMO1 proteins

ProtParam revealed that molecular weights of JUNO and IZUMO1 proteins are 27.93 kDa and 38.89 kDa, respectively. As shown in Figure S3, the signal peptide region at the N-terminus of JUNO and the anchoring region at the C-terminus had strong hydrophobic regions, among which leucine at position 235 was the most hydrophobic (the highest score is 2.5); in the mature peptide region, hydrophobic amino acids and hydrophilic amino acids were evenly distributed. The signal peptide region at the N-terminus and the transmembrane region at the C-terminus of IZUMO1 had strong hydrophobic regions, of which the IIe at position 319 had the strongest hydrophobicity (the highest score was 3.567); hydrophobic and hydrophilic amino acids were also evenly distributed in the mature peptide region.

#### Advanced structural prediction of JUNO and IZUMO1 in Small Tail Han sheep

**Fig. 5:**
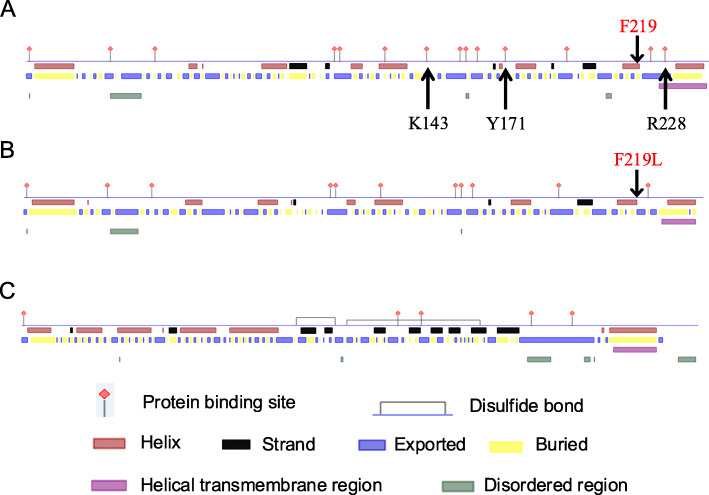
Predicted secondary structure of JUNO and IZUMO1 proteins in Small Tail Han sheep.** a** Predicted secondary structure of JUNO before the mutation at the g.848253C > A locus, based on the full-length *Juno* cDNA of Small Tail Han sheep. **b** Predicted secondary structure of JUNO after the mutation at g.848253C > A, based on the full-length *Juno* cDNA of Small Tail Han sheep. **c** Predicted secondary structure of IZUMO1, based on the full-length *Izumo1* cDNA of Small Tail Han sheep

The secondary structures of Small Tail Han sheep JUNO and IZUMO1 proteins were predicted (Fig. 5). Mutation of the *Juno* gene at the g.848253C > A locus caused an amino acid change from Phe to Leu at position 219. The predicted secondary structures showed there were 14 protein binding regions in the JUNO protein before the mutation at g.848253C > A. After the mutation, three protein binding regions (143, 171, and 228) were lost. There were only five protein binding regions in the predicted secondary structure of IZUMO1, based on the full-length *Izumo1* cDNA of Small Tail Han sheep. But there were two disulfide bonds in the IZUMO1 protein.

**Fig. 6:**
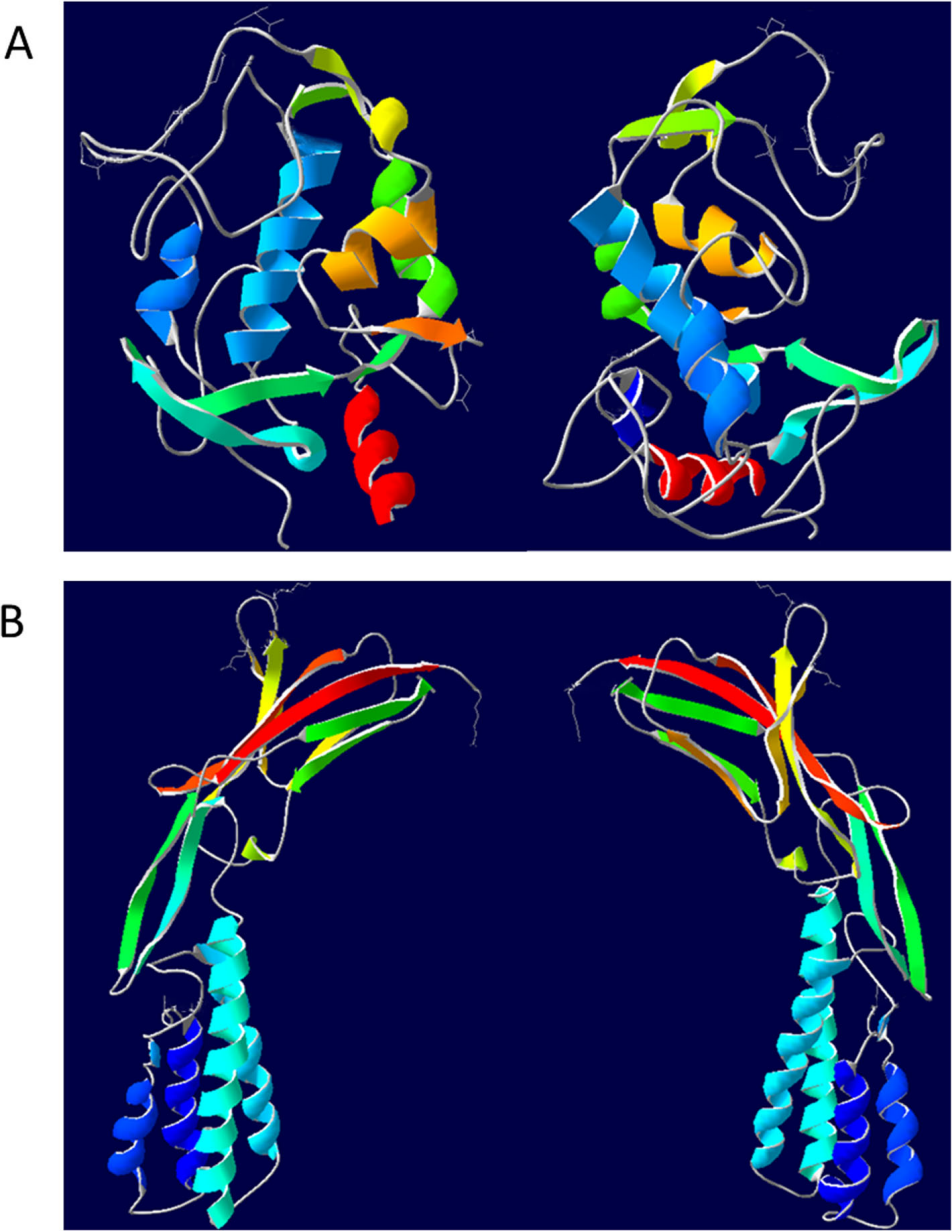
Predicted 3D structures of JUNO (**a**) and IZUMO1 (**b**) from Small Tail Han sheep

Phyre2 was used to analyze the tertiary structures of JUNO and IZUMO1 from Small Tail Han sheep (Fig. 6). The tertiary structure of JUNO was clustered with human oocyte surface protein JUNO, having 75% similarity, whereas it had only 62% similarity to folate receptor α. The tertiary structure of Small Tail Han sheep IZUMO1 was rod-shaped, and had 74% similarity with human sperm IZUMO1.

#### Sequence alignment and phylogenetic analysis of JUNO and IZUMO1 proteins

**Fig. 7:**
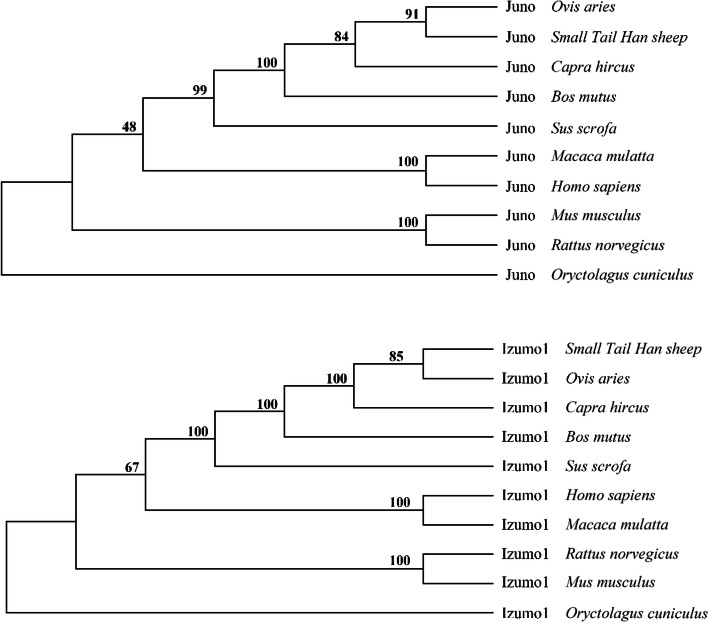
Phylogenetic analysis of JUNO and IZUMO1 proteins in Small Tail Han sheep

The homology between JUNO and IZUMO1 protein orthologs was analyzed. The results showed that the amino acid sequence of JUNO and IZUMO1 in Small Tail Han sheep were conservative among sheep, goat and wild yak. The similarity was much lower with wild boar, human, rhesus monkey, rabbit, rat and mouse (Table 4). A phylogenetic tree was constructed for JUNO and IZUMO1 using the Neighbor-Joining method (Fig. 7). Small Tail Han sheep and sheep (*Ovis aries*) first gathered into one branch, then gathered together with goat (*Capra hircus*).

**Table 4:** The homology between protein orthologs

Species		JUNO		IZUMO1	
		Accession number	Similarity of amino acid sequence with Small Tail Han sheep	Accession number	Similarity of amino acid sequence with Small Tail Han sheep
Sheep	*Ovis aries*	XP_027834903.1	99.6%	NP_001157964.1	99.7%
Goat	*Capra hircus*	XP_017914424.1	97.1%	NP_001274164.1	98.7%
Wild yak	*Bos mutus*	XP_005907338.1	95.5%	XP_014333597.1	95.3%
Wild boar	*Sus scrofa*	XP_013834737.2	79.8%	XP_003127333.3	75.6%
Human	*Homo sapiens*	NP_001186135.1	70.4%	NP_872381.2	65.9%
Monkey	*Macaca mulatta*	NP_001180734.1	69.4%	NP_001181552.1	65.3%
Rabbit	*Oryctolagus cuniculus*	XP_017204775.1	71.2%	XP_017195294.1	54.3%
Rat	*Rattus norvegicus*	XP_017451453.1	63.8%	NP_001017514.1	54.8%
Mouse	*Mus musculus*	NP_075026.1	60.9%	NP_001018013.1	49.6%

We also compared the amino acid sequences of JUNO proteins from different species. We found that compared with human and mouse JUNO proteins, the mutated amino acid (F219L) in Small Tail Han sheep was located in α5 region of human JUNO protein [12] andα7 region of mouse JUNO protein [18], which was closed to GPI signal region, but not belong to residues that interact with Izumo1 and the other three flexible regions.

## Discussion

### The cloning of *Juno* and *Izumo1* genes

Research on the *Juno* gene has mainly focused on its role in mouse and human tumors [19–21], whereas research on its roles in reproduction is relatively rare. At present, there is no complete report of the *Juno* gene sequence in sheep, and there is no full-length cDNA sequence of the *Juno* gene in Small Tail Han sheep, a polytocous sheep breed famous in China. In this study, the complete DNA and cDNA sequences of the Small Tail Han sheep *Juno* gene were successfully obtained. The phylogenetic tree showed that the Small Tail Han sheep JUNO protein had the closest relationship with goat and sheep; the similarity was over 96%, indicating the JUNO protein has been highly conserved throughout evolution.

After identification of the IZUMO1 protein by Inoue et al. [1], many studies have been done on the mammalian *Izumo1* gene, but the cloning and analysis of its cDNA sequence have mostly focused on human and mouse, with a couple of exceptions. Xing et al. [14] cloned and obtained the cDNA sequence of the *Izumo1* gene of the cashmere goat and sheep (breed unknown). Evaluation of the sequences revealed a typical ORF of 963 bp that could be spliced into 8 exons. Kim et al. [13] cloned and obtained the complete ORF sequence of the porcine *Izumo1* gene using RACE technology. We found that most of the currently published *Izumo1* cDNA sequences contained a 963-bp ORF, encoding a 320-amino acid protein, which is consistent with the results of this study, showing that the amino acid sequence of IZUMO1 is relatively conserved among species. Although the *Izumo1* cDNA sequence has been reported in sheep, the breed is unknown and there is no full-length cDNA sequence for Small Tail Han sheep. Previously, we were the first to clone the full-length *Izumo1* DNA sequence of Small Tail Han sheep [17], and in this study, we obtained the first full-length *Izumo1* cDNA sequence of sheep. Alignment of the Small Tail Han sheep *Izumo1* CDS with the sequence from Texel sheep (the reference genome) revealed Small Tail Han sheep *Izumo1* contains an additional segment of CDS that is 35 bp in length. Furthermore, we compared the DNA and cDNA sequences of *Izumo1* from all sheep and found that the *Izumo1* gene has nine exons, not eight as was previously thought. This study provides the basis for future research of the *Izumo1* gene.

The amino acid homology analyses showed that Small Tail Han sheep IZUMO1 had 95% or higher similarity with sheep, goat and cattle. The phylogenetic tree showed that the IZUMO1 protein of Small Tail Han sheep was closely related to that of sheep, goat and cattle, indicating that the IZUMO1 protein has been relatively conserved throughout evolution in Bovidae.

### Expression of *Juno* and *Izumo1* genes

Oocytes are produced in the ovary and mature there. They next enter the oviduct where they await fertilization by the sperm, and time is limited. Once fertilized, oocytes travel to the uterus for implantation. Therefore, the oocyte content in oviduct and uterus is relatively low and fluctuates greatly with time. Transcriptomic data of 40 different sheep tissues showed that expression of *Juno* in lymph node mesenteric, lymph node prescapular and peyer’s patch was the highest, followed by that in ovarian follicles [22]. *Juno* expression in the ovary was slightly lower than in ovarian follicles, while expression in the uterus and corpus luteum was very low [22]. The quantitative results of the present study showed that Juno mRNA was highly expressed in ovary, but weakly or not at all expressed in the oviduct, uterus and other tissues, consistent with transcriptomic data. From the RT-PCR result the Juno had a highly expressed in oviduct, but we believed it might be because of the accuracy of this assay. In order to solve this problem, qPCR and single cell RNA-seq were performed to quantify the expression of Juno precisely.

In this study, single cell RNA-seq data showed that expression of *Juno* was very high in oocytes, but very low in granulosa cells and follicle theca. *Juno* was specifically expressed in oocytes in ovarian tissue, which is consistent with the high expression of *Juno* gene in ovarian follicles in tissue transcriptomic data [22]. It can also be inferred that *Juno* is specifically expressed in oocytes, but not in other ovary cell types. The specific expression of *Juno* in oocytes is related to its function as a receptor during fertilization.

*BMPR1B*, *BMP15* and *GDF9* are all family members of TGF-β pathway. They are also major regulatory genes for litter size in sheep [23]. GDF9 and BMP15 produced by oocytes bind to the BMPR1B receptor located on granulosa cells and act through BMPR1B to jointly stimulate proliferation of granulosa cells [24], which is critical for normal follicular development in sheep. Whether in human or sheep, *GDF9* and *BMP15* have the same expression pattern in follicles [25, 26]. Our single cell RNA-seq data also proved that *BMP15* and *GDF9* are highly expressed in oocytes, whereas *BMPR1B* was highly expressed in granulosa cells. Our RNA-seq data was very consistent with previous results. Like *BMP15* and *GDF9*, *Juno* was specifically expressed in oocytes. However, in both single cells and tissue samples, there was no difference in the expression of *Juno* in sheep differing in litter size, indicating that expression of *Juno* is not related to litter size.

Satouh et al. has firstly described the exposure of IZUMO1 precisely. They visualized the dynamics of diffusion of IZUMO1 at the time of the acrosome reaction using a fluorescent protein tag. IZUMO1 localized in the equatorial segment of the sperm surface after the acrosome reaction [27]. Inoue et al. used a polyclonal antibody prepared by recombinant IZUMO protein for immunolabeling and found that the size of IZUMO in mice is 56.4 kDa, and that it is only expressed in the testes of mice [1]. Kim et al. also confirmed that IZUMO1 is only exposed on the sperm head during sperm-oocyte fusion [13]. Sperm is produced in the testes and then stored in the epididymis. Only when ejaculating will sperm pass through the sperm duct; thus, sperm content in sperm duct should be relatively low. Our data showed that there is a low level of expression in the epididymis, which may be due to the storage of some mature sperm in epididymis.

### The structure and function of JUNO and IZUMO1 proteins

JUNO and IZUMO1 proteins play an important role in the process of sperm and oocyte fusion. The JUNO-IZUMO1 recognition system has been deciphered by X-ray crystallography [12, 28]. After JUNO-IZUMO1 recognition, the dimer IZUMO1 may also specifically recognize a second unknown receptor on the oocyte surface. Sperm-oocyte fusion is a multi-molecule event, and there may be many other molecules involved. Research showed that recognition and binding of JUNO and IZUMO1 is a relatively weak adhesion [29]. After the sperm enters the zona pellucida, IZUMO1 adheres to JUNO, and CD9 on the oocyte accumulates between cells before sperm and oocyte fusion. CD9 was considered to be a protein cooperating with JUNO [30]. This adhesion is conserved in both human and mouse oocytes [10]. Moreover, this kind of adhesion may be transient, needs a sensitive detection method, such as highly sensitive interaction assay [8]. It is also well established that after the first adhesion event between JUNO and IZUMO1, JUNO is released out of the oocyte membrane. Forty minutes after sperm and oocyte fusion, JUNO can longer be detected on the surface of the oocyte membrane. The shedding of JUNO protein is a gradient reaction, which is completed within 40 min. The shedding mechanism of JUNO is closely related to the prevention of multiple sperm from entering the oocyte, ensuring the oocyte will only fuse with one sperm under natural conditions [11].

The tertiary structure prediction of JUNO showed that it has a compact structure with a pocket in the center, which may play an important role in the recognition and binding of ligands or receptor proteins [18]. The tertiary structure prediction of IZUMO1 from Small Tail Han sheep had two stable β-hairpins in the central region of the tertiary structure. Moreover, the α-helix domain in the front end and the immunoglobulin domain in the back end are firmly bound by a disulfide bond to form a slender rod-shaped protein, similar to the tertiary structure of human IZUMO1 [12, 28]. Ohto et al. established that the disulfide bond formed in the β-hairpin region of the IZUMO1 protein is very important to maintain its structural stability [28].

The mutation of the *Juno* gene at locus g.848253C > A will lead to a change from Phe to Leu at site 219 in the amino acid sequence. This single amino acid difference causes a loss of three protein binding sites in JUNO. Interestingly, there was a significant association between the g.848253C > A locus and litter size of Small Tail Han sheep: the litter size of the AA genotype was significantly smaller than the litter size of the CC and CA genotypes. In the 3rd parity, the litter size of sheep with the AA genotype decreased by nearly 1.38 offspring compared with the CC genotype, and there was no difference in litter size between CC and CA genotypes. The latest research showed that four SNPs of the *Juno* gene were found in women with fertilization failure and polyspermy, and variation of *Juno* may have played a role in this pathogenesis [31]. If compare with human and mouse, this mutated amino acid (F219L) in Small Tail Han sheep was not belong to motifs that interact with Izumo1. At present, we don’t know the specific function of this region, but it can be seen that this amino acid is essential for the functionality of JUNO protein in sheep. More functional analysis will be performed to make our doubt clearly in our future research.

## Conclusions

In this study, we successfully obtained the full-length DNA sequence of the *Juno* gene, screened nine SNPs of *Juno* and genotyped these loci. The association analysis showed a significant association between different genotypes of the g.848253C > A locus and litter size of Small Tail Han sheep. The full-length cDNA sequences of *Juno* and *Izumo1* were obtained and 35 bp of new *Izumo1* CDS sequence of in Small Tail Han sheep was found, and we confirmed the *Izumo1* gene has nine exons, not eight exons. Expression analysis showed that the *Juno* gene was highly expressed in ovary, whereas the *Izumo1* gene was highly expressed in testes. In addition, expression of the *Izumo1* gene in the testes of polytocous Small Tail Han sheep was significantly higher than that in monotocous Sunite sheep, indicating that expression of *Izumo1* in testes positively correlates to fecundity. Single cell RNA-seq showed *Juno* is specifically expressed in oocytes, but not in granulosa cells and follicular theca, while *Izumo*1 was barely expressed in all three cell types. There was no difference in the expression of *Juno* in oocyte and ovarian tissue in sheep with different litter size, indicating expression of *Juno* is not related to litter size traits. Bioinformatic analysis revealed the g.848253C > A locus of *Juno* results in a nonconservative missense point mutation leading to a change from Phe to Leu at position 219 in the amino acid sequence. For the first time, this study systematically analyzed the expression, structure and function of *Juno* and *Izumo1* genes and their encoded proteins in Small Tail Han sheep, providing the basis for further research, unravelling the functional mechanisms of *Juno* and *Izumo1* genes in sheep.

## Supplementary Information

**Additional file 1: Supplementary Materials:** Fig. S1: Electrophoresis of PCR products of the *Juno* gene. Fig. S2: Clone and RACE results of the *Juno* and *Izumo1* genes from Small Tail Han sheep. Fig. S3: Prediction of hydrophobicity in Small Tail Han sheep JUNO and IZUMO1 proteins. Table S1: Primers used for amplifying DNA of the *Juno* gene in sheep, Table S2: Primers used for amplifying cDNA and RT-qPCR of the *Juno* and *Izumo1* genes in sheep. Table S3. SNP loci screening.

## Data Availability

The datasets during and/or analysed during the current study available from the corresponding author on reasonable request.
