# Supplementary material for: Expression, structure and function analysis of the sperm-oocyte fusion genes Juno and Izumo1 in sheep (Ovis aries)
Source: J Anim Sci Biotechnol. 2021 Mar 12;12:37. doi: 10.1186/s40104-021-00548-4 (PMC7953763; doi:10.1186/s40104-021-00548-4)
Supplement: Supplementary file 1 — Additional file 1: Supplementary Materials: Fig. S1: Electrophoresis of PCR products of the Juno gene. Fig. S2: Clone and RACE results of the Juno and Izumo1 genes from Small Tail Han sheep. Fig. S3: Prediction of hydrophobicity in Small Tail Han sheep JUNO and IZUMO1 proteins. Table S1: Primers used for amplifying DNA of the Juno gene in sheep, Table S2: Primers used for amplifying cDNA and RT-qPCR of the Juno and Izumo1 genes in sheep. Table S3. SNP loci screening. [file 40104_2021_548_MOESM1_ESM.docx]

**
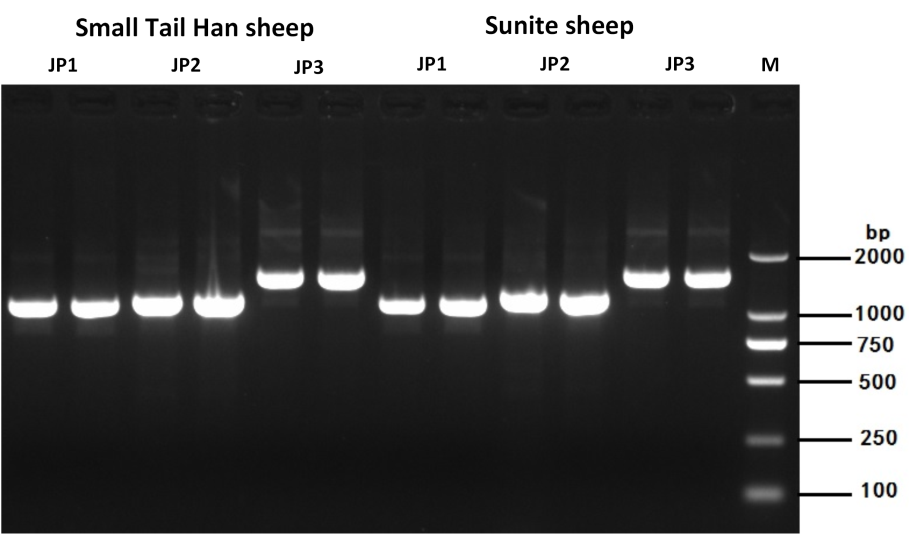
**

**Figure S1.** Electrophoresis of PCR products of *Juno* gene


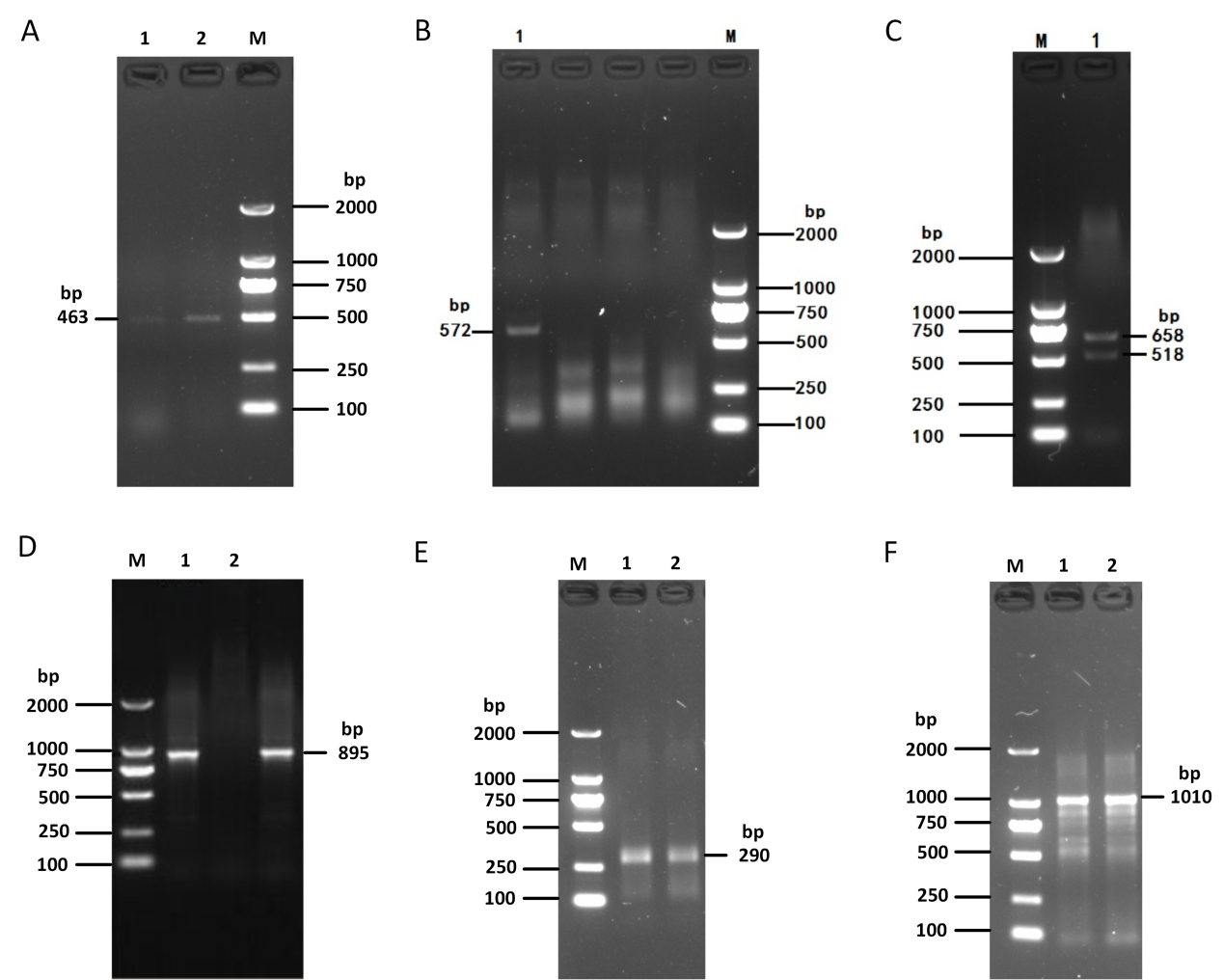


**Figure S2.** Clone and RACE results of *Juno* and *Izumo1* genes of Small Tail Han sheep. (**A**) *Juno* gene partial CDS Clone of Small Tail Han sheep. (**B**) *Juno* gene 3' RACE results of Small Tail Han sheep. (**C**) *Juno* gene 5' RACE results of Small Tail Han sheep. (**D**) *Izumo1* gene partial CDS Clone of Small Tail Han sheep. (**E**) *Izumo1* gene 3 'RACE results of Small Tail Han sheep. (**F**) *Izumo1* gene 5' RACE results of Small Tail Han sheep. Note: M: DL2000 DNA Marker.


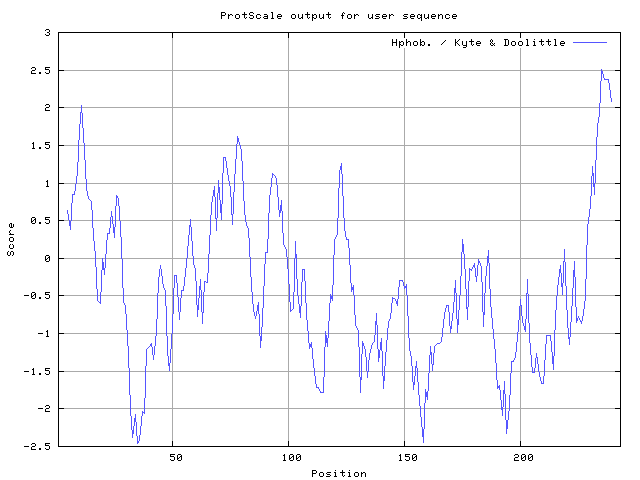


A


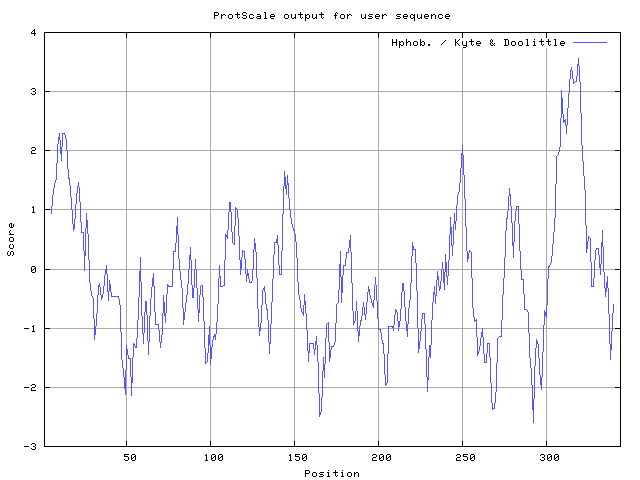


B

**Figure S3**. The hydrophobic prediction in Small Tail Han sheep JUNO and IZUMO1 proteins.**Table S1**. Primers used for amplifying DNA of the *Juno* gene in sheep

| **Primer name** | **Usage** | **Primer sequence (5′ to 3′)** | **Product size, bp** | **Annealing temperature, ℃** |
| --- | --- | --- | --- | --- |
| JP1 | Primer 1 for DNA segment amplification of *Juno* gene | F：TGTGGAGTGAGGGGTTCTGC | 1074 | 66 |
|  |  | R：CAGCAGGCGTTGTCCTTCCA |  |  |
| JP2 | Primer 2 for DNA segment amplification of *Juno* gene | F：ACACCGGCCTATCCCACTTGCA | 1153 | 66 |
|  |  | R：AACCACTTCTGCAGACACCGCC |  |  |
| JP3 | Primer 3 for DNA segment amplification of *Juno* gene | F：TTGTGTGAGCGGATCTGGAGC | 1500 | 66 |
|  |  | R：TAGTTCATGCCTTGGGGATCTGG |  |  |

Note: F means forward primer, R means reverse primer.

**Table S2**. Primers used for amplifying cDNA and real-time PCR of the *Juno* and *Izumo1* genes in sheep

| **Primer Name** | **Primer sequence (5' to 3' )** | **Length, bp** | **Annealing temperature, ℃** |
| --- | --- | --- | --- |
| Juno-CDS | F：GAATGCCAAGCCCCACAAG | 484 | 62 |
|  | R：GATCCGCTCACACAAGTTGG |  |  |
| Juno-RT | F：CACTGCGGACTAATGATGCC | 227 | 60 |
|  | R：CAGTTGGACTTGCAGGTGTG |  |  |
| UPM | CTAATACGACTCACTATAGGGCAAGCAGTGGTATCAACGCAGAGT |  | 68 |
| Juno-3' GSP | CTACTTCCCCACGCCCGCCAACTTGT |  | 68 |
| Juno-5' GSP | GCCGCTCCAGATCCGCTCACACAAGT |  | 68 |
| β-actin | F：CCAACCGTGAGAAGATGACC | 97 | 60 |
|  | R：CCCGAGGCGTACAGGGACAG |  |  |
| Izumo1-CDS | F：GGGTTCTACGACTCCGCTTC | 895 | 58-62 |
|  | R：GGTTACTTTCAGGCCCATCAT |  |  |
| Izumo1-RT | F：ATGCAGGTACCTATCGCTGC | 84 | 60 |
|  | R：TGGGCAATACTGTGACGTGA |  |  |
| Izumo1-3' GSP | TGCTGAGAAGACGCCTGTTCCGAGTGC |  | 68 |
| Izumo1-5' GSP | TGATGGACCTTGACTTCGCGCTCTCC |  | 68 |

**Table S3**. SNP loci screening

| SNP | Variant type |  |
| --- | --- | --- |
| g.844598A>G | 5' UTR | A/G transition |
| g.846206C>T | synonymous SNV | T/C transition |
| g.846268A>G | nonsynonymous SNV | A/G transition |
| g.847219G>C | nonsynonymous SNV | G/C transversion |
| g.847810C>G | nonsynonymous SNV | G/C transversion |
| g.847814C>T | nonsynonymous SNV | T/C transition |
| g.847830T>C | synonymous SNV | T/C transition |
| g.847927A>C | synonymous SNV | C/A transversion |
| g.848253C>A | nonsynonymous SNV | C/A transversion |
